# Protective effect of metformin against retinal vein occlusions in diabetes mellitus – A nationwide population-based study

**DOI:** 10.1371/journal.pone.0188136

**Published:** 2017-11-14

**Authors:** Tai-Chi Lin, De-Kuang Hwang, Chih-Chien Hsu, Chi-Hsien Peng, Mong-Lien Wang, Shih-Hwa Chiou, Shih-Jen Chen

**Affiliations:** 1 Institute of Clinical Medicine, National Yang-Ming University, Taipei, Taiwan; 2 Department of Ophthalmology, Taipei Veterans General Hospital, Taipei, Taiwan; 3 Department of Public Health and Institute of Public Health, National Yang-Ming University, Taipei, Taiwan; 4 National Yang-Ming University School of Medicine, Taipei, Taiwan; 5 Department of Ophthalmology, Shin Kong Wu Ho-Su Memorial Hospital & Fu-Jen Catholic University, Taipei Taiwan; 6 Department of Medical Research and Education, Taipei Veterans General Hospital, Taipei, Taiwan; 7 Institute of Pharmacology, National Yang-Ming University, Taipei, Taiwan; Massachusetts Eye & Ear Infirmary, Harvard Medical School, UNITED STATES

## Abstract

Previous studies have found that metformin can reduce cardiovascular risk, but its association with retinal vein occlusion (RVO) is unknown. In this population-based cohort study using the Taiwan National Health Insurance Research Database (NHIRD), we demonstrated the protective effect of metformin against RVO in diabetes mellitus (DM) and explored the incidence rate and factors associated with RVO development in general and diabetic populations. One million patients were randomly selected from the registry files of the NHIRD, and all their claims data were collected for the 1996–2011 period. Patients with a new diagnosis of central or branch RVO were identified using International Classification of Disease codes. DM was defined for patients with diagnoses and treatments. Factors associated with RVO development in the non-DM and DM cohorts were explored using Cox proportional regression models. In total, 1,018 RVO patients were identified from the database. The average incidence of RVO was 9.93 and 53.5 cases per 100,000 person-years in the non-DM and DM cohorts, respectively. Older age, DM, hypertension, and glaucoma were significant risk factors for RVO, whereas the prescription of anticoagulants was a significant protective factor. In the DM cohort, older age, hypertension, and diabetic retinopathy were significant risk factors for RVO, whereas metformin treatment was a significant protective factor. These results confirmed the risk factors for RVO and demonstrated the protective effect of metformin against RVO in DM patients. Prescribing metformin for DM patients may be beneficial for reducing the incidence of RVO, along with its hypoglycemic action.

## Introduction

Retinal vein occlusion (RVO) is the second most common retinal vascular disease and a major cause of vision loss worldwide. Clinical manifestations of RVO include retinal hemorrhage, congested veins, lipid extravasation, macular edema, optic disk edema, retinal ischemia, and neovascular glaucoma [1]. The 15-year cumulative incidence was estimated to be 2.3% in the United States in 2005 [2]. A study combining individual-level data from 15 major population-based studies on different ethnic groups worldwide reported a summarized prevalence of 4.0 and 0.8 per 1,000 persons for branch and central RVOs, respectively [3]. Although recognized as a disease since 1855, the pathogenesis and management of RVO remain controversial [4]. Risk factors for RVO include old age, diabetes mellitus (DM), hypertension, hyperlipidemia, cardiovascular disease (CVD), and glaucoma [5–7]. Various treatments for RVO have been advocated over the past decade, including laser photocoagulation, thrombolytic agent administration, surgical intervention, and intravitreal steroid or anti-vascular endothelial growth factor (VEGF) agent injection [8, 9]. Among these, anti-VEGF agent injection significantly reduces macular edema, improving visual acuity in most cases [8, 9]. However, these intraocular medications impose a considerable burden on health care systems because of the required repeated administrations, and fewer than 10% of patients have significant vision loss even after multiple anti-VEGF agent injections [10].

DM is associated with several microvascular and macrovascular complications [11]. Hyperglycemia is recognized as a major factor in the pathogenesis of diabetic vasculopathy [12]. According to the United Kingdom Prospective Diabetes Study (UKDPS), microvascular complications of DM are directly related to the severity and duration of hyperglycemia, and intensive glycemic control significantly reduces the risk of microvascular complications [13]. However, the beneficial effect of intensive glycemic management on macrovascular complications remains controversial [14]. Moreover, the UKDPS demonstrated that even when different drugs have the same effects on glycemic control, their effects on CVD can be different [15]. Trials of extremely intensive glycemic management have not consistently shown preventative effects against complications and have even demonstrated some harmful effect in a few cases [16, 17]. Thus, factors other than hyperglycemia may contribute to cardiovascular risk in patients with DM [18, 19]. Recent trials have also demonstrated that hypoglycemic agents had direct cardiovascular effects in addition to the glucose lowering effects [20].

RVO is a peripheral vascular occlusive disease, sharing risk factors, such as carotid artery plaque, hypercoagulable states, and anticoagulant protein deficiencies, with CVD [7, 21]. In DM patients, microvascular and macrovascular complications frequently coexist. Similar mechanisms and shared risk factors drive the development and progression of both small and large vessel disease [22]. However, the association between RVOs and hypoglycemic agents remains unknown. The Taiwan National Health Insurance (NHI) program was introduced in 1995. It is a comprehensively mandatory medical care system organized by the Taiwan government. More than 99% of residents and medical utilities are covered by the NHI program. The present population-based cohort study was conducted to explore the epidemiology and the associated factors leading to RVO development in DM patients by analyzing the National Health Insurance Research Database (NHIRD).

## Materials and methods

### Study design

This population-based retrospective cohort study was designed to analyze the factors associated with RVO development in a representative Chinese population from the NHIRD. This study was approved by the Institutional Review Board of Taipei Veterans General Hospital and adhered to the tenets of the Declaration of Helsinki.

### Setting and participants

The NHIRD is a reimbursement database, in which all claims and registry data associated with medical expenditures of all citizens in Taiwan were collected. In a subset of this database, one million registered beneficiaries were randomly selected using linear congruential random number generation method. These subjects represented for 4.5% of the total population of Taiwan in 2000. All medical claims and registered information between 1997 and 2011 were collected. Diagnoses were defined using International Classification of Disease, Ninth Revision, Clinical Modification (ICD-9-CM) codes, and medical prescription details were extracted from the claims data.

### Inclusion and exclusion criteria

All data between 1997 and 2011 of the selected cohort with or without medical claims were analyzed and reviewed. Patients with abnormal registry claims data, such as unknown sex and inconsequent birthday, were excluded. Patients who died or withdrew from the insurance system before 2001 were not enrolled in the study. Patients with any diagnosis of DM and RVO in their claims data before January 1, 2001, were also excluded, that is, no prior diagnosis between January 1, 1997 and December 31, 2000. In addition, patients with DM were excluded if their claims data contained an RVO diagnosis registered before their DM diagnosis.

### Definition of DM and main outcomes

Patients were defined as having DM if more than three consensus diagnoses (ICD: 250.XX) and corresponding prescriptions of hypoglycemic agents were noted in the database. The first date of the claims data with the DM diagnostic code was considered the DM index date. Patients were then followed until the first central RVO (ICD: 362.35) or branch RVO (ICD: 362.36) diagnosis or until the final entry in the database. Patients without a DM diagnosis (no-DM cohort), were followed from January 1, 2001, until the date of RVO diagnosis or the final entry in the database.

### Exposure to DM medication and metformin

The usage of hypoglycemic medication were identified and classified into eight groups based on the detail prescription claims: metformin, repaglinide, thiazolidinedione, buformin, sulfonylurea, alpha-glucosidase inhibitor, nateglinide, and insulin. As we evaluate the risks for developing RVO in all participants, DM patients were divided into two groups: with or without metformin treatment based on their metformin intake. Metformin intakes of DM patients were expressed into average dosage (mg/day), which was calculated as total prescribed dose divide by total number of follow-up days. Patients who were prescribed metformin with average dose more than 250mg per day were categorized as “with metformin treatment”.

### Other variables

Patient age was stratified into four groups: <35, 35–50, 50–65, and >65 years. The urbanization levels of participants’ registry area was classified into three categories based on population density, medical resources, average age and education level of the people in these areas. According to these criteria, 70 regions in Taiwan were defined as urban, 144 regions were suburban, and 96 regions were rural area.

Other comorbid diseases (e.g., hypertension, hyperlipidemia, coronary artery disease, glaucoma, and diabetic retinopathy) and exposure to comedications (e.g., diuretics, angiotensin-converting enzyme inhibitors, angiotensin receptor blockers, beta blockers, calcium channel blockers, anticoagulants, and fenofibrates) were defined if more than three consecutive diagnostic ICD codes or prescriptions were identified before RVO diagnosis.

### Data analysis and statistical methods

To explore risk factors, and the interaction between drugs for the developing RVO in DM patients, we performed multivariate analysis with 4 stepwise models. First, we adjusted age, usage of anticoagulant therapy, comorbidity of hypertension, glaucoma and diabetic retinopathy. In the second model, the usage of metformin was furtherly analyzed. The usage of repaglinide was added in model three. In the last model, all kinds of anti-diabetic medication were analyzed simultaneously.

Univariate and multivariate analyses for calculating the adjusted hazard ratio (aHR) for the incidence of RVO were performed using Cox proportional regression models. The statistical package SAS statistical package (SAS System for Windows, Version 9.3.1, SAS Institute Inc., Cary, NC) was used for all estimations. The 95% confidence intervals (CIs) of the aHRs were calculated, and all tests were two-sided with the significance level set at 0.05.

### Sensitivity analysis

To avoid possible bias, the association of RVOs and metformin intake was re-analyzed using cumulative method, in which patients were defined as being treated with metformin for more than 2 months. On the other hand, sensitivity analyses were performed to confirm the association between metformin usage and central or branch RVOs.

## Results

**Fig 1:**
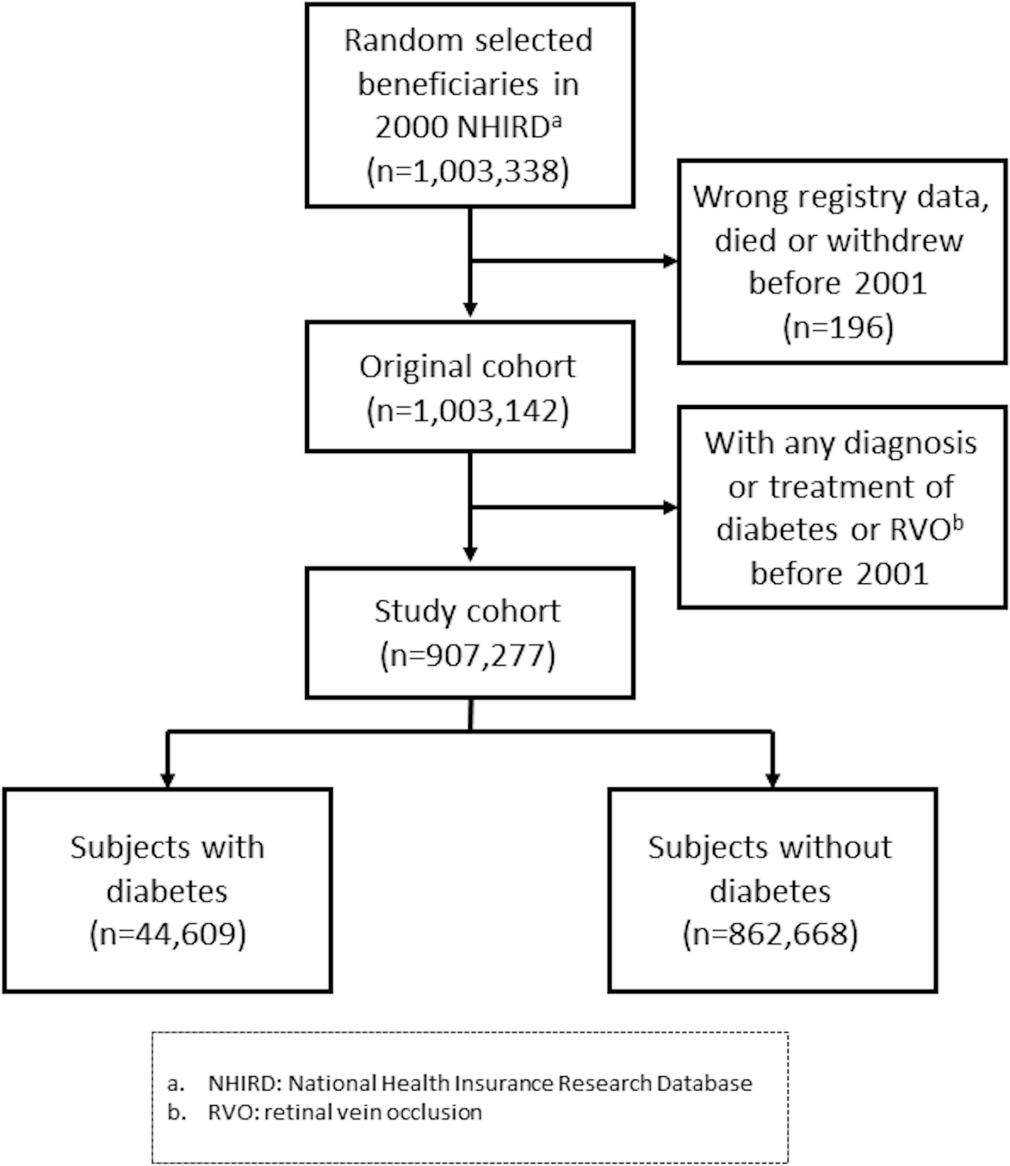
Flow diagram of beneficiaries selection and follow-up. Numbers of individuals at each stage of study.

There were totally 1,003,338 beneficiaries randomly selected, 907,277 of them fully met the inclusion and exclusion criteria, their age ranged from 0 to 104 years in 2001. At the end of 2011, 830,332 subjects remained in the insurance system, with the average follow-up time of 10.4 years. (Fig 1)

The DM and non-DM cohorts comprised 44,609 and 862,668 patients. Among them, 125 DM patients developed RVO (average incidence, 53.5 cases per 100,000 person-years; central RVO, 61 patients; branch RVO, 74 patients) and 893 non-DM patients developed RVO (average incidence, 9.93 cases per 100,000 person-years; central RVO, 298 patients; branch RVO, 625 patients) during the follow-up period. The general characteristics of the patients with RVO are summarized in Table 1.

**Table 1:** Characteristics of patients with retinal venous occlusions in the Taiwan National Health Insurance Research Database.

Characteristics	BRVO^a^ (n = 648)		CRVO^b^ (n = 370)		Control^c^ (n = 906,259)		*p*
**Age (Mean±SD)**	57.5	±12.3	57.5	±14.4	31.9	±19.7	<0.001
**Gender**							0.30
**Male (n, %)**	316	(48.8%)	199	(53.8%)	461,151	(50.9%)	
**Female (n, %)**	332	(51.2%)	171	(46.2%)	445,108	(49.1%)	
**Urbanization of insurance area**							<0.01
**Urban**	368	(56.8%)	215	(58.1%)	541,968	(59.8%)	
**Sub-urban**	196	(30.2%)	123	(33.2%)	283,925	(31.3%)	
**Rural**	84	(13.0%)	32	(8.6%)	80,366	(8.9%)	
**Diabetes (n, %)**	61	(9.4%)	64	(17.3%)	44,484	(4.9%)	<0.001
**Hypertension (n, %)**	441	(68.1%)	232	(62.7%)	166,215	(18.3%)	<0.001
**Hyperlipidemia (n, %)**	103	(15.9%)	62	(16.8%)	60,041	(6.6%)	<0.001
**Coronary artery disease (n, %)**	171	(26.4%)	106	(28.6%)	72,024	(7.9%)	<0.001
**Glaucoma (n, %)**	81	(12.5%)	71	(19.2%)	17,915	(2.0%)	<0.001
**Under anti-coagulant therapy (n, %)**	204	(31.5%)	112	(30.3%)	128,556	(14.2%)	<0.001

^a^ BRVO: branch retinal vein occlusion

^b^ CRVO: central retinal vein occlusion

^c^Control: Patients without branch or central retinal vein occlusion; Patients with both BRVO and CRVO were included in the CRVO group.

Among the study cohort, 648 subjects developed newly diagnosed branch RVO and 370 developed central RVO. Comparing with those who did not have RVO, subjects with RVO were elder and are at higher risk of comorbidities including: DM, hypertension, hyperlipidemia, coronary artery diseases, glaucoma, and receiving anti-coagulant therapy. On the other hand, patients with central RVO were more likely to have the history of DM and glaucoma comparing to those with branch RVO. (*p* <0.001 and <0.01, respectively, data not shown)

Among all patients, according to the multivariate analysis, those with older age, DM with or without metformin treatment, hypertension, or glaucoma more commonly developed RVO during the follow-up period (Table 2); by contrast, patients prescribed anticoagulants had a significantly lower risk for RVO (aHR = 0.65; p < 0.001). Fifty seven percent of the DM patients were prescribed of more than 250mg daily dose of metformin.

**Table 2:** Univariate and multivariate analyses for retinal vein occlusion development.

Characteristics	N(%)	HR^a^	*p*-value	aHR	CI^b^ (upper)	CI (lower)	
**Non-diabetic population**	862,668(95.1)	1 (Reference)		1 (Reference)			
**Diabetic patients without metformin treatment**	18,971 (2.1)	7.04	<0.001	***2.00	1.53	2.61	
**Diabetic patients with metformin treatment**	25,638 (2.8)	5.20	<0.001	***1.61	1.24	2.10	
**Age: age <35 year-old**	538,295(59.3)	1 (Reference)		1 (Reference)			
**Age: 35 ≤ age < 50 year-old**	213,686(23.6)	12.80	<0.001	***9.75	7.22	13.16	
**Age: 50 ≤ age < 60 year-old**	93,664(10.3)	49.24	<0.001	***28.16	20.79	38.14	
**Age: age > 65 year-old**	61,632 (6.8)	69.05	<0.001	***34.94	25.42	48.01	
**Urbanization of insurance area (urban)**	542,551(59.8)	1 (Reference)		1 (Reference)			
**Urbanization of insurance area (sub-urban)**	284,244(31.3)	1.06	0.44	1.04	0.91	1.20	
**Urbanization of insurance area (rural)**	80,482 (8.9)	1.38	<0.01	1.03	0.84	1.26	
**Gender: Male/Female**	461,666(50.9)	1.01	0.87	1.07	0.95	1.22	
**Hypertension (Yes/No)**	166,888(18.4)	9.81	<0.001	***2.48	2.12	2.89	
**Hyperlipidemia (Yes/No)**	60206 (6.6)	3.02	<0.001	0.87	0.73	1.03	
**Coronary Artery Disease (Yes/No)**	72,301 (8.0)	4.84	<0.001	1.03	0.88	1.22	
**Under anti-coagulant therapy (Yes/No)**	128,872(14.2)	2.95	<0.001	***0.65	0.55	0.76	
**Glaucoma (Yes/No)**	18,067 (2.0)	9.03	<0.001	***2.98	2.50	3.56	

Cox proportional regression models were used for the analyses

Patients were defined to be under metformin treatment if their average daily metformin dose was ≥250mg/day

^a^ HR: hazard ratio

^b^ CI: confidence interval * *p* < 0.05, ** *p* < 0.01, *** *p* < 0.001

Compared with non-DM patients, patients with DM had a significantly higher risk of RVO if they were not treated with metformin (aHR = 2.00 and 1.61 if average metformin prescriptions were <250 mg and ≥250mg/day, respectively). These findings were consistent when factors associated with the development of branch or central RVO were explored (S1 Table).

DM patients had a 3.7-times higher central RVO risk if they were not treated with metformin; nevertheless, this declined to only a 2.4-times higher risk with metformin treatment. DM patients also had a 1.6-times higher branch RVO risk if they were not treated with metformin; this incidence declined to that of the non-DM patients with metformin treatment. We also performed a sensitivity analysis by using the cumulative dosage definition of metformin exposure, and all findings were similar.

To confirm the protective effects of metformin, we further performed a multivariate analysis in all DM patients with different models (Table 3) and obtained three main findings. First, after adjustment for all comorbid diseases and comedications, only old age, hypertension, and diabetic retinopathy were significant risk factors for RVO in the DM patients. However, the relationship between RVO and anticoagulant therapy or glaucoma was nonsignificant in the DM patients. Second, the protective effects of metformin were significant and the aHR remained steady in all three models with regard to other hypoglycemic agents (aHR, 0.41–0.46 in Models 2, 3, and 4). Third, another protective effect was observed with regard to repaglinide (aHR = 0.34 in Model 4). However, the protective effect was not observed in DM patients treated with repaglinide but never with metformin (aHR = 0.75, *p* > 0.05), and this effect was stronger for DM patients treated with both drugs (aHR = 0.09, *p* < 0.001). In addition, patients treated with buformin had a high but weak statistically significant risk of RVO, which disappeared in the stepwise analysis.

**Table 3:** Multivariate analysis for retinal vein occlusion development in diabetes mellitus patients.

Variables	Model 1		Model 2		Model 3		Model 4	
	aHR	95%CI	aHR	95%CI	aHR	95%CI	aHR	95%CI
**Age: age <35 year-old**	1 (Reference)		1 (Reference)		1 (Reference)		1 (Reference)	
**Age: 35 ≤ age < 50 year-old**	*3.80	1.17–12.29	*3.95	1.22–12.79	*3.97	1.23–12.87	*3.97	1.22–12.95
**Age: 50 ≤ age < 60 year-old**	**4.88	1.50–15.87	**4.97	1.53–16.18	**4.96	1.52–16.17	*4.72	1.44–14.58
**Age: age > 65 year-old**	**5.49	1.61–18.71	**5.16	1.51–17.63	**5.20	1.52–17.76	*4.80	1.34–3.97
**Hypertension (Yes/No)**	**2.31	1.34–3.95	**2.33	1.36–3.99	**2.31	1.34–3.96	**2.31	1.34–3.97
**Under anti-coagulant therapy (Yes/No)**	0.97	0.64–1.47	0.99	0.65–1.49	1.01	0.67–1.53	1.02	0.68–1.55
**Glaucoma (Yes/No)**	1.51	0.90–2.54	1.48	0.89–2.49	1.45	0.86–2.43	1.46	0.87–2.45
**Diabetic Retinopathy (Yes/No)**	***4.42	3.02–6.45	***4.61	3.15–6.75	***4.98	3.40–7.29	***5.38	3.65–7.95
**Treatments: Metformin/Other drugs**			***0.41	0.27–0.64				
**Treatments: Neither metformin nor repaglinide**					1 (Reference)			
**Treatments: Metformin only**					***0.46	0.30–0.73		
**Treatments: Repaglinide only**					0.75	0.23–2.50		
**Treatments: Both metformin and repaglinide**					***0.09	0.03–0.26		
**Treatment: Metformin (Yes/no)**							***0.46	0.30–0.71
**Treatment: Repaglinide (Yes/no)**							**0.34	0.16–0.73
**Treatment: Thiazolidinedione (Yes/no)**							0.67	0.40–1.12
**Treatment: Buformin (Yes/no)**							^&^3.60	1.12–11.51
**Treatment: Sulfonylurea (Yes/no)**							0.80	0.50–1.29
**Treatment: Alpha-glucosidase inhibitor (Yes/no)**							0.62	0.38–1.01
**Treatment: Nateglinide (Yes/no)**							0.22	0.03–1.58
**Treatment: Insulin (Yes/no)**							0.71	0.32–1.57

aHR: Hazard ratios were adjusted for other covariates, such as urbanization of insurance area, sex, history of hyperlipidemia and coronary artery diseases, and comedication (including diuretics, ACE inhibitors, angiotension receptor blockers, beta-blockers, calcium channel blockers, and fenofibrate), all of which were included in the models

^&^ *p* for buformin = 0.03 * *p* < 0.05, ** *p* < 0.01, *** *p* < 0.001

## Discussion

In this study, we found that the risk of RVO was decreased in DM patients taking metformin as one of their hypoglycemic agents (aHR decreased from 2.00 to 1.61). This effect was more prominent for central RVO. Metformin is a biguanide used as a first line hypoglycemic agent in patients with DM. In addition to glycemic control, metformin can potentially reduce cardiovascular risk [23]. A subgroup analysis in the UKDPS demonstrated a positive cardiovascular impact on DM patients with initial metformin therapy [24]. Studies have revealed that metformin use is associated with lower mortality in DM patients with atherothrombosis as well as with a lower risk of stroke and atrial fibrillation [25–27]. RVO is closely related to CVD [28]. Cardiovascular risk factors, such as atherosclerotic and thrombophilic factors, have been reported for their potential involvement in RVO [6, 21]. In addition to the cardiovascular impact reported in the previous studies, metformin has shown a protective effect against RVO in DM patients in our study; however, the underlying mechanism remains unclear.

Enhanced platelet aggregation contributes to CVD development, particularly in DM patients. Abnormalities in platelet function may exacerbate the progression of atherosclerosis and thrombus formation [11, 29–31]. Hypercoagulability can also lead to RVO because the coagulation system and platelets closely interact during the pathogenesis of thrombosis [6, 32]. In contrast to the results of a population-based study [5] and a study of Hayreh et al. [33], our results show that the intake of either aspirin or other anticoagulants is effective for reducing the risk of RVO in the non-DM population. However, in the DM patients in our study, this effect was not observed, which is in agreement with a subgroup analysis conducted by Hayreh et al. [33], probably because of platelet dysfunction in these DM patients, who became resistant to aspirin [29, 30]. Furthermore, the blood coagulation status of these patients changed with the increase in the number of coagulation factors [34].

Metformin has the favorable effect of inhibiting platelet aggregation and activation, independent of their hypoglycemic effects [23, 35]. Studies have demonstrated that metformin not only limits platelet activation but also improves oxidative stress, preserves antioxidant function, protects endothelial cells, and reduces mean platelet volume in DM patients [25, 35, 36]. A previous study reported that in DM patients, who were resistant to aspirin could reverse the antithrombotic status when they switch to antioxidant therapy [31]. Thus, combining antiplatelet aggregation and oxidative stress improvement in DM patients may be the mechanism underlying the metformin-mediated reduction in RVO risk.

However, some studies have reported contradictory effects of metformin on platelet function [23]. In an experimental study of hypercholesterolemic rabbits that were fed metformin, their platelet response to the aggregating agents was similar to that observed in a group of controls; however, metformin had no effect on the platelet cyclooxygenase pathway [37]. Another study showed that when metformin and another oral hypoglycemic agent were equally effective in achieving glycemic control, these two drugs had similar effects on platelet function [38]. In the present study, hypoglycemic agents other than metformin did not reduce the RVO risk; however, the NHIRD does not contain information on the efficiency of glycemic control.

In the present study, aging was the most significant risk factor for RVO, which is in agreement with the Beaver Dam Eye Study and the Blue Mountains Eye Study [2, 39]. Hypertension and glaucoma were also found to be significant risk factors for RVO in the present study and other studies [5, 6]. To the best of our knowledge, no population-based data of the incidence of RVO in DM patients have been reported. Our data reveal an average incidence of 0.53 cases per 1,000 person-years in the DM cohort and 0.1 cases per 1000 person-years in the non-DM cohort. Previous case–control studies have demonstrated a weak and inconsistent association between RVO and DM [40, 41]. The Beaver Dam Eye Study showed that the prevalence of branch RVO is associated with DM [42]. A study on various ethnic groups demonstrated that RVO is associated with a higher prevalence of DM in Asians and West Indians than in Caucasian Europeans [41]. Our study confirms aging and hypertension are associated with RVO incidence in DM patients.

In our DM cohort, we observed that diabetic retinopathy is significantly associated with RVO incidence, which is in contrast to the results of a previous small-scale case–control study [43]. In DM patients, retinopathy morbidity reflects DM severity and its association with CVD [44]. However, in previous clinical trials of anti-VEGF therapy for RVO, patients with any evidence of diabetic retinopathy on examination were excluded [45–47]; consequently, the association between RVO and diabetic retinopathy remained unknown. The present study is the first to demonstrate that diabetic retinopathy is a potential risk factor for RVO.

Our result show that repaglinide treatment alone had no protective effect against RVO in DM patients, but a protective effect was noted when repaglinide was combined with metformin (aHR = 0.09, *p* < 0.001); this protective effect was more pronounced than that in those treated with metformin alone. Thus, repaglinide and metformin can synergistically reduce the cardiovascular risk in DM patients [48]. However, the exact mechanism for the synergistic effect of repaglinide and metformin on lowering RVO risk remains unclear: whether the combination therapy improves glycemic control or cardiovascular parameters remains unknown [48].

The major limitation of the present study is the lack of clinical data. Some crucial factors, such as blood sugar levels, serum cholesterol and lipid levels, and body weight, are unavailable in the NHIRD. Thus we could not precisely adjust the models for DM severity without clinical data. Furthermore, we do not know whether patients who switched to or added on a different class of hypoglycemic drugs other than metformin may reflect a poor glycemic control. Therefore, we used the presence of diabetic retinopathy as an indicator for DM severity in this study. Differences between the DM and non-DM cohort, such as age, health activity, and diet habit would exist and lead to some selection bias. Thus we have adjusted all available confounders using various statistical models. In addition, the pathophysiology of central or branch RVO may be slightly different. Hence we separately performed the sensitivity analysis for central and branch RVO, and the protective effect of meformin is significantly demonstrated in both types of RVO. Finally, patients with asymptomatic RVOs or those with symptoms but did not seek medical assistance might not have been recorded, leading to an underestimated RVO incidence. However, the probability of this underestimation was likely similar in both the DM and the non-DM cohort.

## Conclusion

Our results reveal the incidence of and risk factors for RVO in a population-based cohort and the protective effect of metformin against RVO in patients with DM. Although the exact mechanism remains unclear, prescribing metformin for DM patients may be beneficial for reducing the incidence of RVO, along with its hypoglycemic action. Additional clinical and experimental studies should be conducted to confirm our findings.

## Supporting information

S1 Table Multivariate analyses for development of branch retinal vein occlusion and central retinal vein occlusion. Cox proportional regression models were used for analyses. Patients were defined to be under metformin treatment if their average daily metformin dose was ≥250mg/day ^a^ BRVO: branch retinal vein occlusion; ^b^ CRVO: central retinal vein occlusion. * *p* < 0.05, ** *p* < 0.01, *** *p* < 0.001.
(DOCX)
