# Supplementary material for: Protective effect of metformin against retinal vein occlusions in diabetes mellitus – A nationwide population-based study
Source: PLoS One. 2017 Nov 14;12(11):e0188136. doi: 10.1371/journal.pone.0188136 (PMC5685597; doi:10.1371/journal.pone.0188136)
Supplement: S1 Table — Cox proportional regression models were used for analyses. Patients were defined to be under metformin treatment if their average daily metformin dose was ≥250mg/day a BRVO: branch retinal vein occlusion; b CRVO: central retinal vein occlusion. * p < 0.05, ** p < 0.01, *** p < 0.001. (DOCX) [file pone.0188136.s001.docx]

**Supplemental Table 1. Multivariate Analyses for Development of Branch Retinal Vein Occlusion and Central Retinal Vein Occlusion**

|  | | | | | | |
| --- | --- | --- | --- | --- | --- | --- |
| **Characteristics** | **BRVO^a^** | | | **CRVO^b^** | | |
|  | **aHR** | **CI-** | **-CI** | **aHR** | **CI-** | **-CI** |
| **Non-diabetic population** | **1 (Reference)** |  |  | **1 (Reference)** |  |  |
| **Diabetic patients without metformin treatment** | ****1.61** | **1.14** | **2.26** | *****3.66** | **2.53** | **5.32** |
| **Diabetic patients with metformin treatment** | **1.33** | **0.95** | **1.86** | *****2.38** | **1.58** | **3.60** |
| **Age: age <35 year-old** | **1 (Reference)** |  |  | **1 (Reference)** |  |  |
| **Age: 35 ≤ age < 50 year-old** | *****12.42** | **8.35** | **18.49** | *****6.15** | **3.97** | **9.51** |
| **Age: 50 ≤ age < 60 year-old** | *****36.65** | **24.56** | **54.71** | *****17.19** | **11.05** | **26.73** |
| **Age: age > 65 year-old** | *****39.79** | **26.17** | **60.48** | *****27.31** | **17.18** | **43.39** |
| **Urbanization of insurance area (urban)** | **1 (Reference)** |  |  | **1 (Reference)** |  |  |
| **Urbanization of insurance area (sub-urban)** | **1.05** | **0.89** | **1.24** | **1.07** | **0.86** | **1.34** |
| **Urbanization of insurance area (rural)** | **1.15** | **0.91** | **1.46** | **0.76** | **0.52** | **1.12** |
| **Gender: Male/Female** | **1.04** | **0.90** | **1.21** | **1.20** | **0.98** | **1.48** |
| **Hypertension (Yes/No)** | *****2.72** | **2.26** | **3.27** | *****1.97** | **1.52** | **2.57** |
| **Hyperlipidemia (Yes/No)** | **0.84** | **0.68** | **1.04** | **0.84** | **0.63** | **1.14** |
| **Coronary Artery Disease (Yes/No)** | **1.00** | **0.82** | **1.21** | **1.22** | **0.93** | **1.60** |
| **Under anti-coagulant therapy (Yes/No)** | *****0.68** | **0.56** | **0.83** | *****0.58** | **0.44** | **0.76** |
| **Glaucoma (Yes/No)** | *****2.52** | **2.01** | **3.16** | *****4.08** | **3.12** | **5.35** |
|  |  |  |  |  |  |  |

Cox proportional regression models were used for analyses

Patients were defined to be under metformin treatment if their average daily metformin dose was ≥250mg/day

^a^ BRVO: branch retinal vein occlusion; ^b^ CRVO: central retinal vein occlusion

* p < 0.05, ** p < 0.01, *** p < 0.001
